# Anomaly of Haughton type D left cervical aortic arch in combination with type B dissection: case report and literature review

**DOI:** 10.1186/s13019-018-0768-8

**Published:** 2018-06-27

**Authors:** Alicja Zientara, Igor Schwegler, Nicolas Attigah, Michele Genoni, Omer Dzemali

**Affiliations:** 1 Department of Cardiac Surgery, Triemli Hospital, Birmensdorferstrasse 497, 8063 Zürich, Switzerland; 2 Department of Vascular Surgery, Triemli Hospital, Birmensdorferstrasse 497, 8063 Zürich, Switzerland

**Keywords:** Cervical aortic arch, Aortic anomaly, Aortic dissection, Haughton classification

## Abstract

**Background:**

The anomaly of cervical aortic arch is a rare phenomenon first described by Reid in 1914 and categorized by Haughton in 1975. The left cervical aortic arch Type D consisting of an ipsilateral descending aorta and coarctation or aneurysmatic formation of the arch demonstrates a complicated form requiring surgical management. Because of its rarity and unspecific symptoms only few cases are documented with the focus on surgical management.

**Case presentation:**

A 43-year old, asymptomatic woman presented with a mediastinal mass overlapping the aortic arch region in a routine x-ray. For verification, a computed tomography was performed and revealed incidentally a type B dissection originating from an aneurysm of a left cervical arch with a maximum diameter of 6 cm. Because of the huge diameter and the potential risk of rupture, an urgent surgical repair was planned. Surgical access was performed through median sternotomy and an additional left lateral thoracic incision through the fourth intercostal space. Simultaneously to the preparation, partial cardiopulmonary bypass was installed in the left groin. After preparation of the recurrent and phrenic nerve and the supraaortic branches, the descending aorta was clamped. Before the distal anastomosis to a straight graft, we performed a fenestration of the dissection membrane about a length of 5 cm to preserve the perfusion of both lumina. Then, the straight graft was sutured to the proximal part of descending aorta. The left axillary artery originated directly from the aneurysm and was dissected and reimplanted with a separate 8 mm sidegraft to the straight graft between the distal arch and proximal descending aorta. The patient was extubated on first postoperative day and recovered well.

**Conclusion:**

The left cervical aortic arch type D is a rare disease, which is prone to aneurysm formation due to abnormal flow patterns and tortuosity of the aorta. The difficulty lays in the identification of the pathology, especially in the physical examination, since a pulsating mass or cervical murmur seem to be the most specific symptoms in the majority of young, female patients. If diagnosed, surgical therapy with resection of the aneurysm and reimplantation of the axillary artery under cardiopulmonary bypass demonstrates the treatment of choice.

## Background

In 1975, Haughton published the classification of the cervical aortic arches and distinguished between five different types (Haughton type A-E) respecting the configuration of the aorta, the sequence of brachiocephalic branching and embryogenesis [1]. Moreover, Haugthon documented 25 cases of cervical aortic arches, of which four showed a type D configuration. In the same year, Moncada et al. published their review focusing on 23 cases and the association with cardiac anomalies [2]. Compared to further configurations of cervical arches, it is noticeable that the type D arches primarily demonstrate a surgical challenge. It is characterized by an ipsilateral descending aorta, a normal sequence of brachiocephalic branching and a coarctation of the arch leading to a potential risk of development of an aneurysm. Cardiac anomalies do not tend to be associated with this type of aortic anomaly. A correlation with the occurrence of aortic dissection has never been reported since our recent publication of images of a 33-year old woman in 2014 [3]. Most aortic arch aneurysms of Haughton type D occur between the left common carotid and left axillary artery [4]. Analyzing the 44 patients, who are documented since 1968 in the current literature, the left cervical aortic arch is diagnosed mainly in younger women in their 20ties or 30ties. The symptoms range from a painless pulsating mass to dyspnea and dysphagia. Three cases are described as ruptured aneurysms requiring emergency treatment [5–7]. We analysed 39 publications describing 44 cases of type D cervical arch [2, 4–40] (Table 1). Based on this analysis, we describe the experience and therapy of our case. Because of the aneurysm formation in young patients, the type D cervical arch is an essential pathology to be discussed in a surgical forum. Facing its rarity and the lack of a standardized therapy, a summary of surgical interventions and outcome might be helpful for the treatment of this unusual disease.

**Table 1:** Published cases of left cervical aortic arch Haughton type D with aneurysm and/or coarctation: LCA (left carotid artery), LAA (left axillary artery), CPB (cardiopulmonary bypass)

Publication	Gender/Age	Symptoms	Aneurysm/Kinking	Procedure	Outcome
Baravelli (2005) [9]	F/38	Dyspnea, Murmur	K	–	–
	M/18	Murmur	K	–	–
Barbee (2007) [33]	M/32	Chest pain	A + K	2-step: endovascular axillary-to-axillary bypass	6-month FU
Camiel (1982) [19]	F/51	Incidental finding on x-ray	A	–	–
Cao (1980) [5]	F/21	Dissecting aneurysm, emergency	A	Dacron prosthesis between LCA and LAA	10th postoperative day
Charrot (2009) [10]	F/48	Progredient pulsatile cervical mass	A	Aneurysm resection, direct anastomosis, LAA reimplantation with graft, CPB	11-month FU
Chen (2002) [11]	F/25	Chest dyscomfort	K	conservatively	–
	F/14	Palpable cervical thrill	A	Dacron prosthesis, LAA reimplantation with graft	3-month-FU
Chen (2009) [23]	F/7	Recurrent pneumonia, dyspnea	no	Operative treatment, Ligamentum arteriosum and right abberant AA, vascular ring	–
Deffrenne (1968) [34]	–	No symptoms	–	–	–
	–	No symptoms	–	–	–
DuBrow (1974) [24]	M/23	Shortness of breath, dizziness	K	Ligation of LAA, Dacron prosthesis, CPB	–
Farsak (1998) [25]	F/24	headache, tinnitus and numbness in left upper limb, dysphagia	A	Prosthesis, LAA reimplantation with graft	6-month FU
Higuchi (2003) [12]	F/16	Pulsating cervical mass	A	Prosthesis, LAA reimplantation with graft, CPB	4-year-FU
Hirao (1999) [20]	M/59	Incidental finding	A	Aneurysm resection, Gelwave Bypass, LAA reimplantation direct	–
Hoshino (1982) [37]	F/24	?	A	Artificial graft replacement, temporary bypass	–
Ikonomidis (1999) [6]	M/23	Left hematothorax, emergency	A + K	Dacron prosthesis, CPB	12th postoperative day
Imai (2000) [13]	M/48	Headache, Murmur	A + K	Dacron prosthesis	–
Ito (2014) [26]	F/57	hoarseness and dysphagia	A + K	Prosthesis, LAA reimplantation	28th postoperative day
Kame (1982) [40]	F/39	Rupture, emergency	A	–	–
Kaul (2013) [31]	M/56	transient loss of consciousness	A	Prosthesis, LAA ligation	4-year-FU
Kazuno (1988) [38]	M/56	?	A	Exclusion, extraanatomic bypass	–
Khoury (2008) [32]	M/53	interscapular pain	A + K	–	–
Kumar (1989) [35]	F/37	?	A	–	–
Lorusso (2006) [14]	F/40	neck pulsation during physical activity	no	conservatively	–
McCue (1973) [15]	F/11	Pulsating mass in the neck	–	–	–
Mitsumori (2008) [21]	F/38	Incidental finding	A	Prosthesis, LAA and LCA reimplantation	32-month FU
Moncada (1974) [2]	−/1	Dyspnea, Dysphagia	–	–	–
	−/2	Cyanosis, Clubbing of digits	–	–	–
Montgomery (1981) [16]	M/23	Pulsating mass, hoarseness	A	Prosthesis	–
Morris (1978) [27]	F/36	Dysphagia	A	conservatively	–
Noguchi (2003) [4]	M/59	transient ischemic attack.	A	Prosthesis Dacron, reimplantation LAA, CPB	Stroke with incomplete right homonymous hemianopia 36th postoperative day
Ogawa (2002) [28]	F/41	Dysphagia	A + K	Operation	–
Ogawa (1994) [39]	F/30	?	A	End-to-end anastomosis	–
Pearson (1997) [8]	F/11	Dyspnea	A	Prosthesis, CPB	6-month FU
	F/6	Hemiparesis, pulsating mass	A	Prosthesis	8-year FU
Pitzus (1974) [36]	F/39	?	A	?	–
Takahashi (2011) [29]	M/59	inspiratory obstruction	A	Trifurcated Graft, CPB	12-month FU
Tsukamoto (2003) [22]	M/58	Incidental finding	A	conservatively	–
Türkvatan (2009) [17]	F/27	pulsatile mass	A	Bypass LCA-Aorta	–
Van Nooten (1986) [30]	F/17	Dyspnea	A	Dacron Graft, LAA ligation	10th postoperative day
Wei (1983) [7]	F/22	?	A + K	Artificial graft replacement	–
Yigitbasi (1971) [18]	F/22	Pulsatile mass	A	–	–
Zientara (2014) [3]	F/33	Back pain	A	Prosthesis, reimplantation LAA, CPB	3-month FU

## Case presentation

**Fig. 1:**
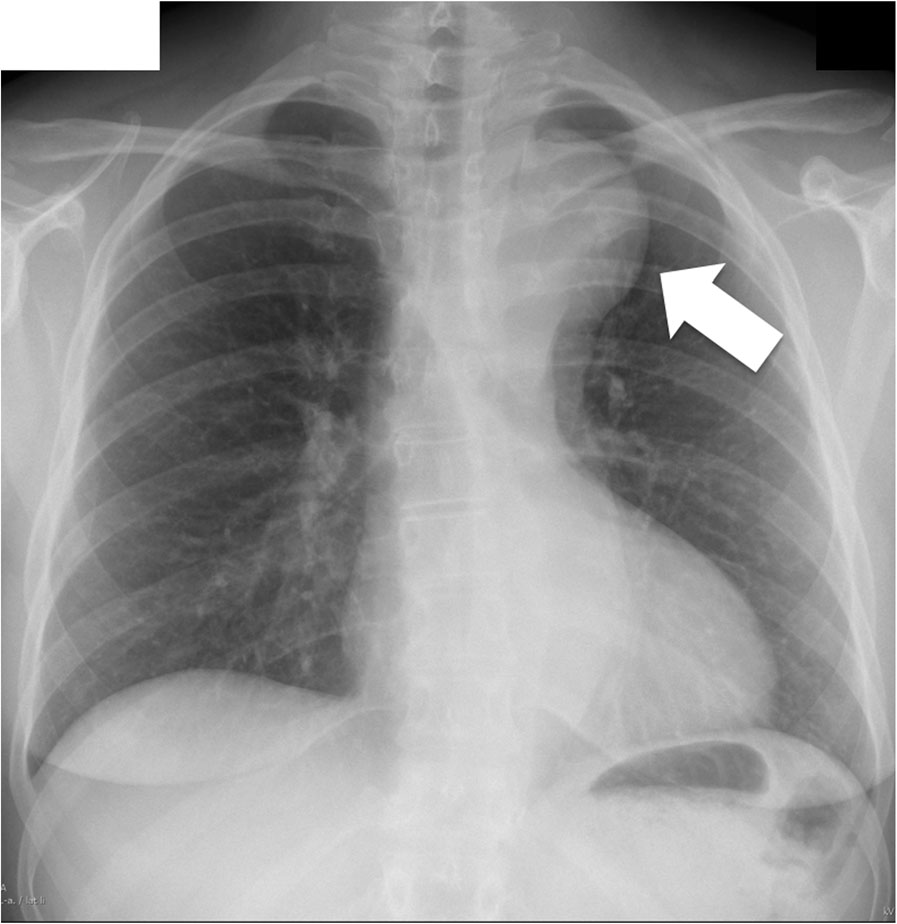
Chest-x-ray with enlarged mediastinum (arrow)

**Fig. 2:**
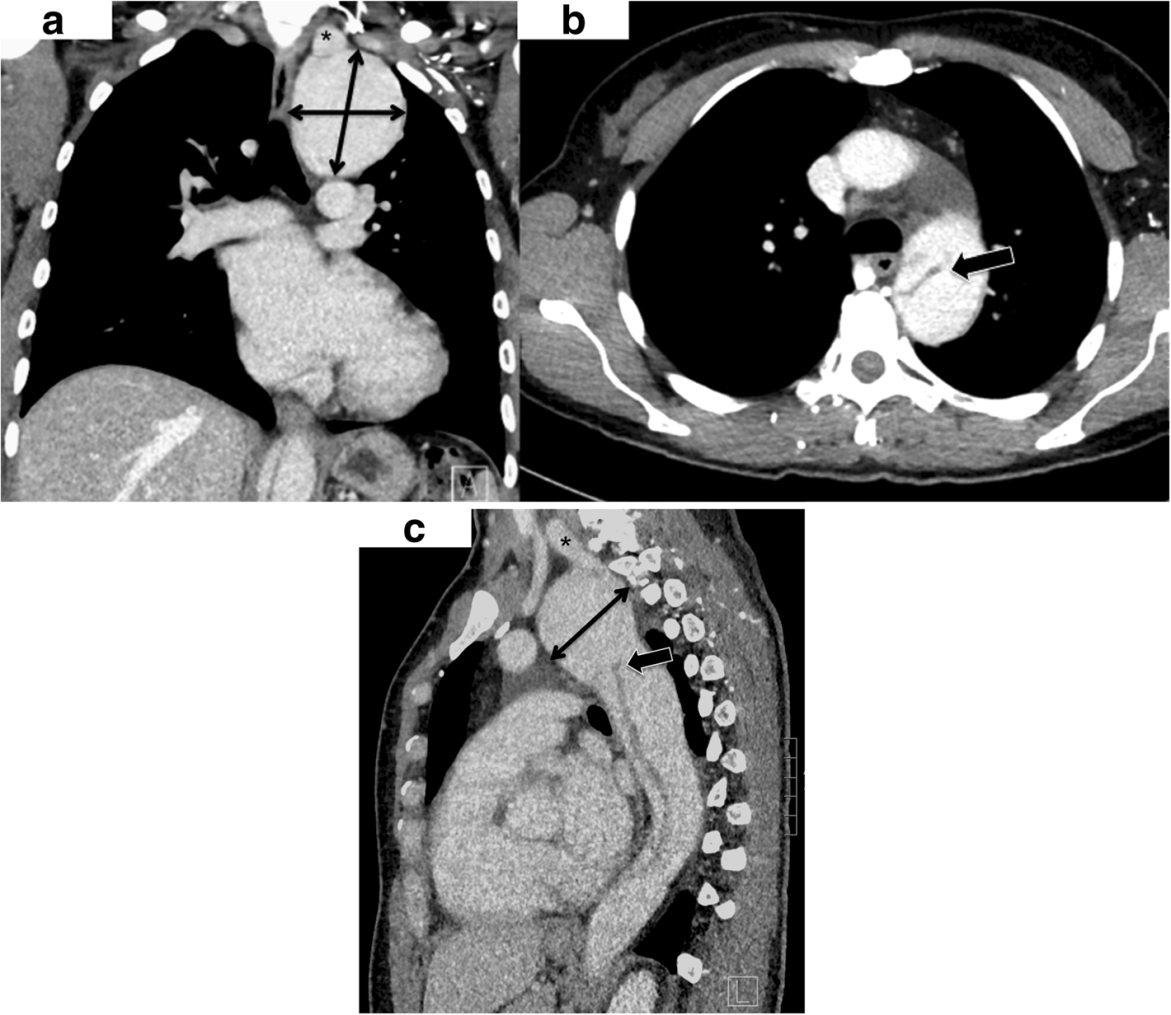
**a** Computed tomography of the cervical arch aneurysm with a maximal extend of 6 cm. * marks the origin of the left axillary artery. **b** Membrane of the type B dissection directly deriving from the aneurysm (arrow). **c** Lateral view with the localisation of the aneurysm in the upper posterior mediastinum. The subclavian artery branches from the top of the aneurysm and runs to the anterior part of the upper mediastinum (*). The dissection membrane starts inside the aneurysm to the abdominal aorta

**Fig. 3:**
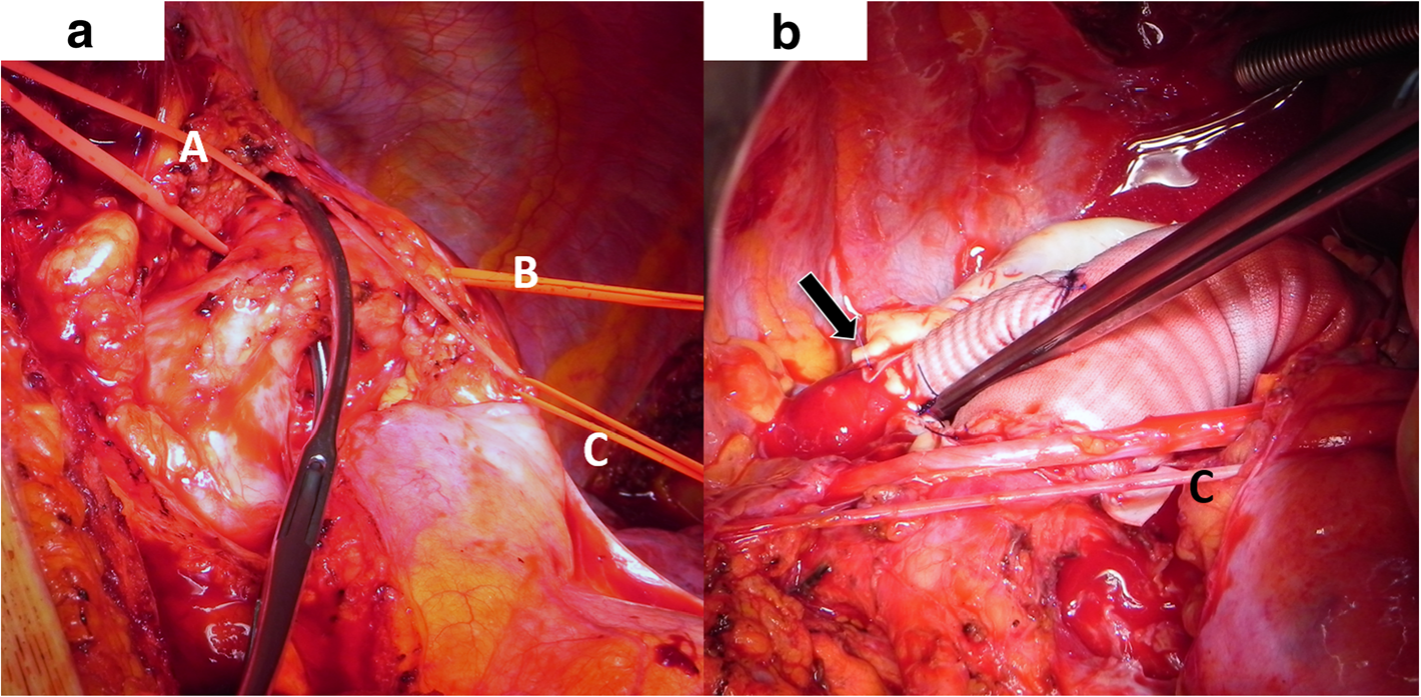
**a** Intraoperative view: Loop A marks the left common carotid artery, loop B marks the left axillary artery. The aortic clamp is positioned between both arteries. Loop C marks the phrenic nerve. **b** Intraoperative view of the end-to-end distal anastomosis of the sidegraft from the main prosthesis to the axillary artery

A 43-year old, asymptomatic woman was admitted to our hospital by her family doctor after receiving a chest-x-ray during routine clinical examination. The x-ray showed a mediastinal mass overlapping the aortic arch region (Fig. 1). For verification a computed tomography (CT) was performed and revealed incidentally a type B dissection, which was most likely chronic without information of the index date, originating from an aneurysm of a left cervical arch with a maximum diameter of 6 cm (Fig. 2a-c). The left renal artery, the coeliac trunc and the main part of the superior mesenteric artery branched from the false lumen without a sign of malperfusion of the organs. Because of the huge diameter and the potential risk of rupture, an urgent surgical repair was planned. Before intervention the patient got a blood pressure adjustment by ACE inhibitor. Betablocker was not necessary because of a resting pulse under 60 beats per minute. For neurological online monitoring, sensitive and motor evoked potentials were monitored. Spinal drainage was installed 1 day before the procedure. Surgical access was carried out through median sternotomy and an additional left lateral thoracic incision through the fourth intercostal space (Hemi-Clemshell). Simultaneously to the preparation of the aneurysm, partial cardiopulmonary bypass was installed in the left groin by cannulation of the femoral artery and vein under echocardiographic guidance. During selective ventilation of the right side, the left lung was mobilized by transsection of the Ligamentum pulmonale and preparation of the perianeurysmatic tissue and adhesions. After identification and preparation of the recurrent and phrenic nerve and the supraaortal branches, the descending aorta was clamped and a distal anastomosis performed with a straight graft (20 mm). The visceral arteries partially branched from the false and true lumen without a sign of malperfusion. Before the final distal anastomosis, we performed a fenestration of the dissection membrane about a length of 5 cm to keep the perfusion of both lumina. The left carotid artery originated from the aortic arch with a distance of only 1 cm from the aneurysm. The left axillary artery branched directly from the aneurysm and was dissected and reimplanted with a separate 8 mm sidegraft to the 20 mm straight graft between the distal arch and proximal descending aorta. (Fig. 3a, b). The procedure was performed with partial cardiopulmonary bypass (CPB) of 87 min, aortic clamp time of 62 min under normothermic condition. The patient was extubated on first postoperative day and recovered well.

Biopsy of aortic tissue showed a picture consistent with arteriosclerosis and loss of smooth muscle cells, rupture of the elastic fibres and fibrosis of the media. The intima could not be visualized in detail.

The patient was discharged to cardiac rehabilitation at 13th postoperative day and recovered well. Last follow up with computed tomography was performed 3, 5 years after initial operation with a good and stable result of the dissection membrane and a perfusion of both lumina. The patient is able to resume a normal life without limitations.

## Discussion

### Data source and literature searches

We searched PubMed/MEDLINE for relevant full-text articles, case reports and abstracts. No restrictions were placed on the language or year of publication. The search included all articles from 1971 to 2014. The following keywords were searched through Pubmed/MEDLINE: “Haughton aortic arch” OR “Haughton type D” OR “aortic arch anomaly” OR “persistent aortic arch” OR “left persistent aortic arch” OR “cervical aortic arch” OR “left cervical aortic arch”.

### Symptoms and diagnosis

The main clinical sign of a left cervical aortic arch (LCAA) aneurysm in the documented patients was a cervical pulsating mass or cervical thrill in asymptomatic patients. The clinical picture includes a murmur and a swelling pulsatile mass at the basis of the neck [8–18]. An incidentally diagnosed LCAA in chest-x-ray or physical examination can be seen in many patients [19–22]. Further signs and symptoms of a compression of neighboring structures, such as stridor, dyspnea, recurrent bronchitis and dysphagia are described in a minority of patients [2, 8, 9, 23–40]. Neurological disorders like hemiparesis or transient ischemic attack resulting from embolism are seldom [4, 8, 31]. In three documented cases an emergency operation due to a rupture of the unknown aneurysm was performed [5–7]. Interscapular pain or back pain like in our first patients’ anamnesis belong to the uncommon symptoms and may lead primarily to the diagnosis of diseases of the vertebral column [32]. Back pain belongs to the typical symptoms of type B dissection as well, but might not be the first hypothesis in younger patients. It is more difficult to establish the diagnosis in patients with unspecific or absent symptoms like in our case [11, 33]. However, most patients remain asymptomatic [9]. The first hint at the correct diagnosis often was a suspicious, enlarged mediastinum on the x-ray. Further investigations including CT or angiography in older publications followed and confirmed the anomaly.

### Interventions and follow up

Due to a number of individual techniques, we summarized an overview of the interventions and the follow up in Table 1.

Only few patients in the documented cases underwent a surgical treatment. Four patients were treated conservatively on the grounds of refusal of operation, oligosymptomatic course and lack of an aneurysm. There is no information about the follow up [11, 14, 22, 27].

In 13 cases the therapy remained unclear [2, 9, 15, 18, 19, 32, 34–36]. In 27 cases an operation was performed with a more or less precise description of the technique. In the majority of the cases the aneurysm was resected and left axillary artery anastomosed to the graft or directly to the aorta. In few cases a direct end-to-end anastomosis of the aorta without a graft because of the kinking was possible. Some authors describe the anastomosis of the axillary artery with the use of an additional graft as mentioned in this present case and our former patient in 2013 [3]. In most cases the procedure was performed with the help of partial cardiopulmonary bypass for perfusion of the visceral arteries and lower body (Tab. 1). One case (m/32y) was managed with an endovascular technique by a thoracic graft covering the left axillary artery [33]. An axillary-to-axillary bypass was necessary at second postinterventional day because of left upper extremity ischemia. In the 6-month follow up the arch aneurysm and the hypoplastic left vertebral artery had thrombosed and the patient recovered well without any symptoms. The condition for an endovascular treatment in our patients was not given because of the visceral perfusion from both lumina and the dissection of both femoral arteries.

The unique feature about our patient, in contrast to a few reports previously published, includes the presence of a type B dissection originating directly from the arch aneurysm. We completed our procedure with the fenestration of the distal aortic membrane to provide the perfusion of the arteries deriving from the false lumen.

Only in 14 operated cases the follow up is documented and ranges from the day of discharge to 4 years. The only patient with a postoperative complication suffered from a stroke and residual incomplete right homonymous hemianopsia [4]. Before, the main symptoms in this case were transient ischemic attacks.

The complication of a mild ptosis as the clinical result of a postoperative Horner syndrome in one patient demonstrates the advanced demands on the surgeons’ preparation because of anatomical changes due to the displacement of structures by the aneurysm or kinking [3].

### Further facts: Genetics and cardiac anomalies

The deletion of chromosome 22q11 and cardiac anomalies have to be mentioned in the context of cervical aortic arches. The deletion of chromosome 22q11 (del22q11) was described in few patients with cervical aortic arches. This syndrome is associated in approximately 75–80% of patients with certain forms of ventricular outflow tract anomalies and ventricular septal defect, dismorphic facial features, aplasia/hypoplasia of the thymus and parathyroid glands, defects of velo-cardial structure, mild mental retardation with speech and behaviour difficulties, renal and skeletal anomalies.

Baravelli et al. confirmed the association between cervical aortic arches and del22q11 in two syndromic patients with dysmorphic features and multiorgan anomalies belonging to the group of right cervical aortic arches with different patterns of supraaortic branches [9]. Both, the association of cardiac anomalies and deletion of chromosome 22q11 could not be found in the current literature of documented cases of LCAA type D.

In our patient, no cardiac anomaly could be diagnosed in the transthoracal or transoesophageal echocardiography. Histological findings of the aortic wall showed mucoid degeneration, which might be associated with fibrillopathy. Further genetic analysis was not performed after reviewing the clinical examination results, which did not speak for a genetic manifestation.

## Conclusion

The left cervical aortic arch type D is a rare disease, which is prone to aneurysm formation due to abnormal flow patterns and tortuosity and redundancy of aorta. It might play a role as potential, life threatening vascular disease. The difficulty lays in the identification of the pathology, especially in the physical examination, since a pulsating mass or cervical murmur seem to be the most specific symptoms in the majority of young, female patients. If diagnosed, surgical therapy with resection of the aneurysm and reimplantation of the axillary artery under cardiopulmonary bypass demonstrates the treatment of choice. Endovascular treatment might be an option in selected patients.

## Abbreviations

ACE inhibitor: Angiotensin converting enzyme inhibitor
cm: Centimeter
CPB: Cardiopulmonary bypass
CT: Computed tomography
LAA: Left axillary artery
LCA: Left carotid artery
LCAA: Left cervical aortic arch
mm: Millimeter
